# Fiber-Optic Pedicle Probes to Advance Spine Surgery through Diffuse Reflectance Spectroscopy

**DOI:** 10.3390/bioengineering11010061

**Published:** 2024-01-08

**Authors:** Merle S. Losch, Justin D. Heintz, Erik Edström, Adrian Elmi-Terander, Jenny Dankelman, Benno H. W. Hendriks

**Affiliations:** 1 Department of Biomechanical Engineering, Faculty of Mechanical Engineering, Delft University of Technology, 2627 CD Delft, The Netherlandsj.dankelman@tudelft.nl (J.D.);; 2 Department of Clinical Neuroscience, Karolinska Institutet, 171 77 Stockholm, Sweden; 3 Capio Spine Center, 115 26 Stockholm, Sweden; 4 Image Guided Therapy and Ultrasound Devices and System Department, Philips Research, Royal Philips NV, 5656 AE Eindhoven, The Netherlands

**Keywords:** Diffuse Reflectance Spectroscopy, spine surgery, breach detection, fiber optics, probe design

## Abstract

Diffuse Reflectance Spectroscopy (DRS) can provide tissue feedback for pedicle screw placement in spine surgery, yet the integration of fiber optics into the tip of the pedicle probe, a device used to pierce through bone, is challenging, since the optical probing depth and signal-to-noise ratio (SNR) are affected negatively compared to those of a blunt DRS probe. Through Monte Carlo simulations and optical phantom experiments, we show how differences in the shape of the instrument tip influence the acquired spectrum. Our findings demonstrate that a single bevel with an angle of $30^{\circ }$ offers a solution to anticipate cortical breaches during pedicle screw placement. Compared to a blunt probe, the optical probing depth and SNR of a cone tip are reduced by 50%. The single bevel tip excels with 75% of the optical probing depth and a SNR remaining at approximately ⅔, facilitating the construction of a surgical instrument with integrated DRS.

## 1. Introduction

**Figure 1:**
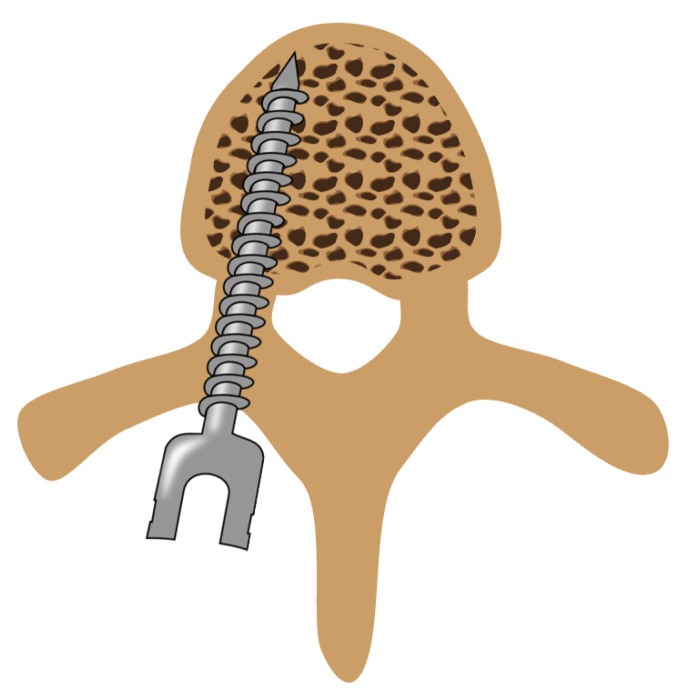
Illustration of a pedicle screw placed inside the vertebra (axial view).

The spine is the central support structure of the human body and provides both flexibility and stability. It consists of individual vertebrae, forming the spinal canal that functions as a protective tunnel for the spinal cord and nerves. Spinal deformities, back pain, tumors, or acute traumatic injury may require medical treatment in the form of spinal fusion [1,2,3,4]. During this surgical intervention, screws may be placed through the bone to connect adjacent vertebrae and ensure mechanical stability. Most commonly, screws are placed through the pedicles (see Figure 1). Accuracy is crucial for successful surgery, as misplaced screws may result in poor stability or cause severe complications such as nerve damage or spinal cord injury. Harming these vital structures can result in pain, loss of sensation, muscle weakness, or even complete paralysis below the level of injury [5,6].

Pedicle screw misplacement frequency using the freehand technique is commonly reported at 10% [7,8]. To improve upon this, real-time tissue feedback could be used to detect impending cortical breaches by probing ahead of the surgical instrument. Optical sensing has emerged as a promising approach for tissue feedback, offering directed measurements to provide insights into tissue composition and structure. In this context, Diffuse Reflectance Spectroscopy (DRS) has been proven to provide tissue feedback for spine surgery in a non-harmful way [9,10,11]. The reflectance spectra result from the interplay of light scattering and absorption by tissue constituents within the probed region. Bone tissue exhibits absorption peaks due to fat, water, and collagen, key absorbers within the wavelength range of interest (visible (VIS) – near infrared (NIR)).

DRS technology is sufficiently small to allow for integration into a variety of surgical devices, including pedicle screws [12] and surgical drills [13]. While these are feasible options, we aim to develop a more practical and effective method to integrate DRS into the surgical workflow by investigating the incorporation of optical fibers into pedicle probes, the main means for pedicle cannulation. Pedicle probes are used to pierce through bone during spinal fusion and typically have a tapered or cone-shaped tip, which poses a challenge for the incorporation of two parallel optical fibers. We explore this challenge by investigating how the optical signal is influenced by an extended, absorbing tip compared to a blunt probe. Moreover, we consider alternatives including a single-bevel design where one fiber is offset along the device axis to match the slant in a protruded position relative to the other fiber. By investigating cone and single-bevel design through Monte Carlo (MC) simulations and optical phantom experiments for different tip angles, we provide insights into the use of DRS during pedicle cannulation that highlight the importance of the tip shape of the pedicle probe.

## 2. Materials and Methods

### 2.1. Monte Carlo Simulations

To provide insight into the volume probed with different cone-shaped tips, MC simulations were run in MCmatlab [14]. In the simulation, photon packets are launched and their trajectories are traced through the simulated volume. As the photon packets propagate, energy is deposited based on the voxels’ absorption coefficient, and scattering events can take place, the path length between and the scattering angle of which are computed pseudorandomly.

**Figure 2:**
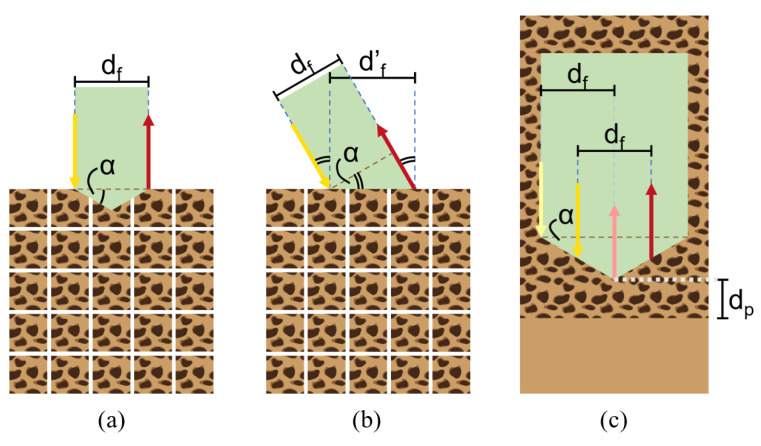
Illustration of the setup for (**a**) the cone simulation, (**b**) the single bevel simulation, and (**c**) the phantom experiment. The pose of the optical probe is indicated in green. The directions and locations of the emitting fibers are indicated with yellow arrows; the directions and locations of the light-collecting fibers are indicated with red arrows.

At λ=1211nm, the wavelength of maximum absorption of fat in the NIR range [15], $10^{9}$ photons were emitted into a single-layer cancellous bone model (absorption coefficient $\mu_{a}\left(1211\;\mathrm{nm}\right)=1.4220$, reduced scattering coefficient $\mu_{s}^{\prime }\left(1211\;\mathrm{nm}\right)=19.5129$, Henyey–Greenstein scattering anisotropy factor g=0.9 [10,11]) with a resolution of 100 bins/mm. The extended tip ($\alpha \;{[}^{\circ }]\in \left\{0,15,30,45,60\right\}$) was assumed to be fully absorbing ($\mu_{a}\left(1211\;\mathrm{nm}\right)=10^{6}$). The scattering coefficient and anisotropy factor of the tip were set to the same values as for cancellous bone ($\mu_{s}^{\prime }\left(1211\;\mathrm{nm}\right)=19.5129$, g=0.9). The setup of the simulation is illustrated in Figure 2a.

To closely replicate the DRS setup, the emitted light was approximated by a top-hat beam. This beam had a focal plane intensity distribution width equal to $1\;/\;e^{2}$ times half the optical fiber core diameter (200 μm), and an angular intensity distribution half-angle equal to $1\;/\;e^{2}$ times the inverse sine of the optical fiber numerical aperture (NA) (0.22). The light collector, on the other hand, was defined as an optical fiber with a core diameter of 200 μm and an NA of 0.22, placed at a distance of $d_{f}=1.4\;\mathrm{mm}$ from the source.

For comparison, the same simulations were run for different single bevel-shaped tips. As MCmatlab does not allow for fibers to be placed inside the simulation volume, both fibers were instead tilted by $\alpha \;{[}^{\circ }]\in \left\{0,15,30,45,60\right\}$, and the fiber distance was adjusted to $d_{f}^{\prime }=d_{f}/\cos\left(\alpha \right)$. The setup of the simulation is illustrated in Figure 2b.

### 2.2. Phantom Experiments

Skeletal bone is composed of a strong and compact outer layer, the cortical bone, and a less dense inner part called cancellous bone. Phantom experiments were conducted to investigate how the tip design affects the measured diffuse reflectance spectrum in the proximity of the cortical bone boundary. For the experiments, a two-layered optical phantom was created, with the bottom layer simulating cortical bone and the top layer simulating cancellous bone. The cortical bone-mimicking layer was created from a mixture of water, NaCl (Groupe Salins, Clichy, France), 15% gelatin (250 bloom porcine gelatin powder, Dr. Oetker, Bielefeld, Germany), barium sulfate (Acros Organics B.V.B.A., Geel, Belgium), and sodium benzoate (Natural Spices B.V., Mijdrecht, The Netherlands). As for the cancellous bone-mimicking layer, pure coconut milk with an 18% fat content (Go-Tan B.V., Kesteren, The Netherlands) was used as a cost-effective alternative to the commonly used Intralipid^®^ 20% IV fat emulsion (Baxter International Inc., Deerfield, IL, USA) [11].

**Figure 3:**
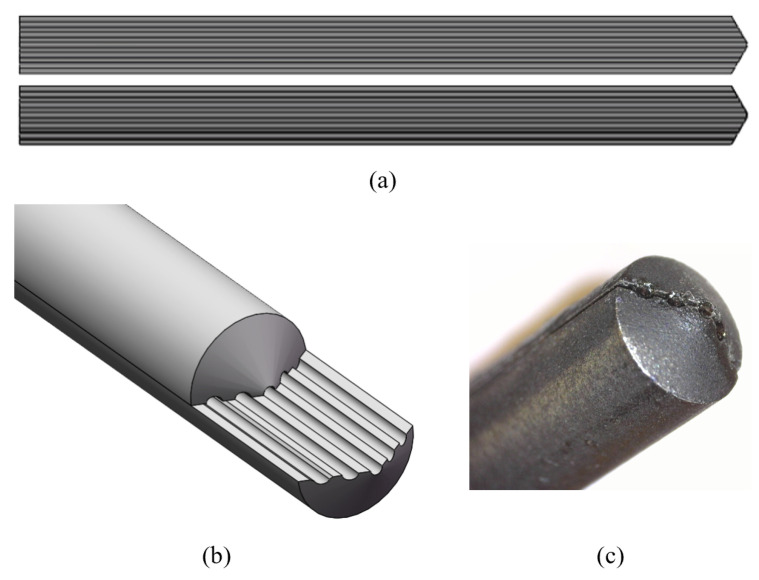
(**a**,**b**) Illustration of the custom-designed optical probe with a tip of $\alpha =30^{\circ }$ and space for two pairs of parallel optical fibers. (**c**) Close-up view of the assembled probe tip.

Based on our MC simulation results and the design of commercially available pedicle probes, we custom designed a probe with a tip of $\alpha =30^{\circ }$ (Figure 3a,b). The experimental setup illustrated in Figure 2c shows how the probe provides space for two pairs of parallel optical fibers (step-index multimode fiber optic patch cables, core diameter 200 μm, NA = 0.22, low OH, Thorlabs Inc., Newton, NJ, USA). The first pair consists of two fibers symmetrically arranged around the tip to emit and collect light to and from the tissue, at a distance $d_{f}=1.4\;\mathrm{mm}$ from each other (cone design); the second pair consists of one fiber at a distance $d_{f}=1.4\;\mathrm{mm}$ from the tip to emit light to the tissue and one fiber at the tip to collect light from the tissue (single-bevel design).

The probe was manufactured from stainless steel and coated with matte black spray paint (RAL 9005 matte black, Cosmos Lac, Athens, Greece). A close-up view of the probe tip with the fibers included is provided in Figure 3c. The light-emitting fibers can be connected to a tungsten halogen broadband light source with an integrated shutter (HAL-S, Avantes, Apeldoorn, The Netherlands). The light-collecting fibers can be connected to a NIR spectrometer with an InGaAs detector (S330-2 NIR, HORIBA Scientific, Piscataway, NJ, USA) to collect light at 255 distinct wavelengths between 839.65 nm and 1724.27 nm. The DRS console runs Philips custom-developed software to control the system. Prior to conducting any measurements, the system underwent calibration using a Spectralon white reference standard (WS-1-SL, Labsphere Inc., North Sutton, NH, USA). The optical probe was positioned perpendicular to the standard at a distance of 3.2 mm, capturing an intensity calibration spectrum to correct for any wavelength-dependent sensitivities in the setup.

For the experiments, the probe was oriented perpendicular to the interface of the two model layers and mounted onto a manual 25 mm linear translation stage with an optical stage position encoder (Thorlabs Inc., Newton, NJ, USA). At various distances to the interface of the two phantom layers $d_{p}\;\left[\mathrm{mm}\right]\in \left\{0,0.25,\ldots ,2.5\right\}$, ten diffuse reflectance spectra were measured in each case, with an integration time of 1000 ms. The experiment was repeated three times at different points within the phantom. For each experiment, the ten spectra recorded at every distance were averaged and filtered with a third-order Savitzky–Golay filter (frame length of 11) to remove noise.

## 3. Results

### 3.1. Monte Carlo Simulations

**Figure 4:**
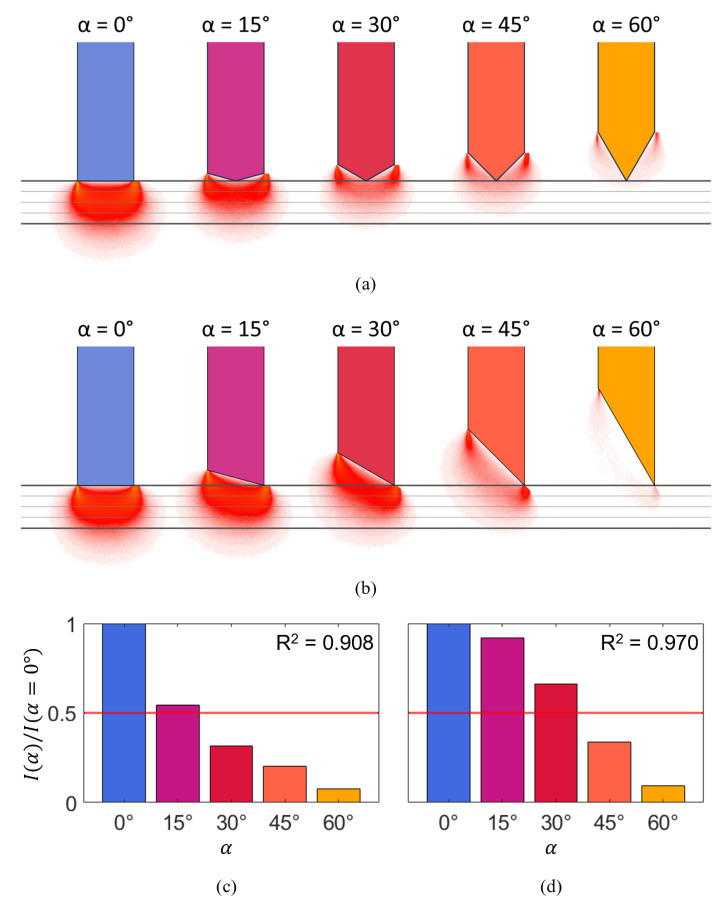
(**a**,**b**) Projected normalized fluence rate of collected photons for different tip angles for (**a**) the cone simulation, and (**b**) the single bevel simulation. The horizontal lines indicate the location of the probe tips and the optical probing depth of the blunt probe for reference. (**c**,**d**) Photon count for different tip angles relative to the photon count observed for $\alpha =0^{\circ }$ for (**c**) the cone simulation, and (**d**) the single-bevel simulation. Red line indicates half of the original photon count.

Figure 4a,b show the normalized fluence rate (the accumulated energy deposited into the voxels as photon packets propagate through the simulation volume, normalized to the input power and divided by the absorption coefficient [14]) of collected photons, orthogonally projected onto the plane of the two fibers for the two tip designs, cone, and single bevel. The probed volume was approximated as the mean photon path ± 1 std, which corresponds to a volume comprising the paths followed by 68.3% of all detected photons. The optical probing depth was defined as the distance between the probe tip and the deepest point to which the probed volume extends, and is indicated in the graph for the blunt probe ($\alpha =0^{\circ }$). To compare the optical probing depth that can be achieved, the projected normalized fluence rates for all tip angles α are plotted next to each other.

With the cone design (Figure 4a), the plot shows that light goes beyond the absorbing probe tip for $\alpha \leq 45^{\circ }$. For a tip angle of $\alpha =15^{\circ }$, the optical probing depth is reduced to approximately 75% of that of the blunt probe. For a tip angle of $\alpha =30^{\circ }$, the optical probing depth has halved. The MC simulations show that not only the optical probing depth but also the total photon count depends heavily on the tip angle, as shown in Figure 4c. For $\alpha =30^{\circ }$, the extended absorbing cone-shaped tip attenuates the obtained signal to well below half the original signal. The total photon count for $\alpha >30^{\circ }$ suggests that the SNR suffers significantly for sharper tips.

With the single-bevel design (Figure 4b), light goes beyond the probe tip for all tip angles. For a tip angle of $\alpha =15^{\circ }$, the optical probing depth is reduced only slightly. For a tip angle of $\alpha =30^{\circ }$, the optical probing depth is still approximately 75% of that of the blunt probe. The probed volume for $\alpha =45^{\circ }$ suggests that photons still reach relatively deep into the tissue, although the total photon count also decreases for higher tip angles (see Figure 4d). While exhibiting the same decreasing trend in signal-to-noise ratio (SNR), unlike the results observed in the cone simulation, the signal obtained with the single-bevel design for $\alpha =30^{\circ }$ experiences considerably less attenuation, remaining at approximately ⅔ of the photon count acquired with the blunt probe. For very sharp tips ($\alpha >45^{\circ }$), in turn, the total photon count reaches similarly low values as for the cone design.

### 3.2. Phantom Experiments

**Figure 5:**
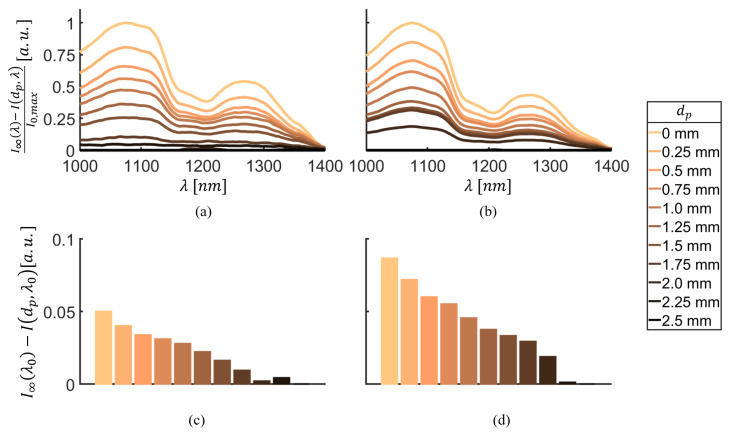
(**a**,**b**) Reflectance spectra for different distances to the interface relative to the reflectance spectrum of the pure cancellous bone-mimicking phantom, normalized to the maximal intensity of the pure cortical bone-mimicking phantom for (**a**) the cone design, and (**b**) the single-bevel design. (**c**,**d**) Intensity at $\lambda_{0}=1211\;\mathrm{nm}$ measured for different distances to the interface relative to the intensity at $\lambda_{0}$ of the pure cancellous bone-mimicking phantom for (**c**) the cone design and (**d**) the single-bevel design.

Figure 5 compares typical reflectance spectra for approaching the interface of the two phantom layers $I(d_{p},\lambda )$ acquired with the two different tip designs. The spectra are plotted relative to the reflectance spectrum $I_{\infty }\left(\lambda \right)$ acquired on the pure cancellous bone-mimicking phantom ($d_{p}\to \infty$) with the respective tip design, recreating a pedicle screw placement scenario where the surgeon first obtains a reference spectrum from cancellous bone. This reference spectrum is then used to monitor the rest of the procedure through continuous measurements, alerting the surgeon about deviations to prevent impending breaches. The spectra are further normalized to the maximal intensity observed at the interface of the two phantom layers $I_{0,max}$, and are plotted for a wavelength range from 1000 nm to 1400 nm because the greatest differences in the signal are to be expected here.

With both the cone design (Figure 5a) and the single-bevel design (Figure 5b), a gradual change in spectrum can be seen between the cancellous bone-mimicking layer ($d_{p}=2.5\;\mathrm{mm}$, dark brown) and the cortical bone-mimicking layer ($d_{p}=0\;\mathrm{mm}$, light beige). While both tip designs yield similar results close to the interface of the two layers ($d_{p}\leq 1.5\;\mathrm{mm}$), a clear difference between the cone design and the single-bevel design can be seen when first approaching the cortical layer ($d_{p}=2.25\;\mathrm{mm}$ and $d_{p}=2.0\;\mathrm{mm}$). With the cone design, the corresponding lines are very close to the pure cancellous spectrum, whereas a clear change in intensity magnitude and shape of the spectrum can be observed for the single-bevel design. Between $d_{p}=2.0\;\mathrm{mm}$ and $d_{p}=1.5\;\mathrm{mm}$ distance to the interface of the two phantom layers, the spectral shape is similar for both designs, yet the intensity is still higher for the single-bevel design.

These findings are supported by the (non-normalized) intensities at $\lambda_{0}=1211\;\mathrm{nm}$ shown in Figure 5c,d. The difference in intensity between the cancellous and the cortical bone-mimicking layers is relatively small with the cone design, as Figure 5c shows. With the single-bevel design, the total difference in intensity between the two phantom layers is approximately double compared to the cone design, see Figure 5d.

## 4. Discussion and Conclusions

Pedicle cannulation requires a pedicle probe with a sharp tip; however, this tip shape poses a challenge for the incorporation of fiber optics as required for DRS. Next to investigating the influence of an extended cone-shaped tip on the optical signal, we therefore consider alternative designs that incorporate a single bevel where one fiber is offset along the device axis, in a protruded position relative to the other fiber. The MC simulation results show that light goes beyond the probe tip (optical probing depth >0) with the cone design for tip angles up to $45^{\circ }$ and with the single-bevel design for tip angles up to $60^{\circ }$. Still, the optical probing depth decreases for sharper tip angles, as does the SNR. Optical probing depth and SNR seem to suffer more from the absorbing probe tip that extends between the two fibers in the cone design than they do from having one fiber protruded relative to the other fiber as in the single-bevel design.

From our simulation results, a tip angle of $30^{\circ }$ seems to be the cut-off up to which reasonably good sensing results can be achieved with the single-bevel design, as it still allows for 75% of the original optical probing depth and ⅔ of the original signal strength. Therefore, we opted for this tip angle for our custom-designed probe, and made it accommodate both tip designs to allow for direct comparison.

The phantom experiments conducted with the custom-designed probe confirm our observations from the simulations that the single-bevel design outperforms the cone design. The difference in signal received between cancellous and cortical bone-mimicking phantom with the cone design was relatively low, supposedly for two reasons: due to the absorption happening at the extended tip, and because for high tip angles, light—following Fermat’s principle—starts traveling around the tip instead of ahead, which alters the probed volume and reflects in the acquired spectrum. This is not the case when one of the fibers is protruded relative to the other fiber and placed at the tip of the pedicle probe. The difference in signal received between cancellous and cortical bone with the single-bevel design was therefore higher, suggesting that optical probing depth and SNR of this design allow for better detection of impending breaches. This is supported by the changes in magnitude and shape of the spectral lines when first approaching the cortical layer. As photons start to travel through both media, absorption and scattering properties change. The first signal changes are visible earlier for the single-bevel design, indicating that this design allows to detect cortical breaches well before they occur.

Two possible pedicle probe designs that arise from these insights are (1) an actual single bevel tip with a diameter of at least the fiber distance, or (2) a cone-shaped tip with a diameter of at least twice the fiber distance that houses one fiber in the tip. The latter would even allow for symmetric designs with a multitude of emitting fibers located around a single collector inside the pedicle probe tip, allowing to further increase the SNR.

An optical pedicle probe can provide surgeons with real-time information on the bone composition inside the vertebra beyond tactile feedback alone. Dynamically redirecting the probe enables exploration of longer trajectories without breaching the cortical boundary, allowing for the insertion of screws with optimal dimensions for superior biomechanical strength, a critical advantage in compromised bone conditions like osteoporosis.

### 4.1. Limitations

This study’s primary limitation is the assumption that the extended tip is fully absorbing. This assumption affects the results for the cone design, where we would expect higher optical probing depth and SNR for a reflecting tip compared to an absorbing tip. In contrast, the optical properties of the tip appear to have limited impact on the single bevel tip’s performance, with little change in the probed volume up to tip angles of $30^{\circ }$. An absorbing probe tip represents the most challenging scenario, indicating that while the cone design is less effective with an absorbing tip, it may still function with a reflecting one. The single-bevel design remains effective for both a reflective and an absorbing tip.

Our study focused on two specific designs for probe tips, the cone and the single bevel. However, the spectrum of possible designs extends beyond these two options. We did not explore probe tips where one fiber is placed on the opposite side of the cone, but closer to the tip (so still protruded relative to the other fiber). While we anticipate such a design to yield results intermediate to the two extremes tested here, it merits further investigation to prove how much light is absorbed at the extended tip, and to what extent light travels around the tip instead of ahead. Although this design cannot compete with the single-bevel design in terms of signal strength and optical probing depth, placing the protruded fiber off of the tip can save some space in diameter if a cone-shaped tip is desired.

We also did not investigate the impact on the optical signal of rounding off the tip of a cone-shaped pedicle probe. Nevertheless, we expect the effect of such a modification to be minor compared to the substantial effect that the single-bevel design can make, in addition to the impairment that a round tip poses for the pedicle probe’s performance.

In this study, we investigated which would be the best pedicle probe from a sensing point of view, but the design of a pedicle probe is primarily dependent on mechanical and surgical factors (e.g., the vertebral level operated on, the condition of the bone tissue in connection with age-related changes and the presence of osteoporosis, and the personal preference of the surgeon). These factors collectively determine the optimal design and must be taken into consideration before the final pedicle probe can be developed to actually promote the integration of DRS into the surgical workflow.

### 4.2. Conclusions

DRS could enhance spine surgery by allowing to detect impending cortical breaches. The aim of this study was to investigate the impact of the tip shape of a pedicle probe on the DRS signal. With our results from MC simulations and optical phantom experiments, we demonstrate that a single bevel with an angle of $30^{\circ }$ is superior to a cone-shaped tip for detecting proximity to the cortical layer. These findings pave the way for future integration of DRS technology into surgical instruments for improved pedicle screw placement accuracy.

## Data Availability

Data underlying the results presented in this paper are available in [16].

## Abbreviations

The following abbreviations are used in this manuscript:

DRS	Diffuse Reflectance Spectroscopy
MC	Monte Carlo
NA	Numerical aperture
NIR	Near infrared
SNR	Signal-to-noise ratio
